# Do specific delirium aetiologies have different associations with death? A longitudinal cohort of hospitalised patients

**DOI:** 10.1007/s41999-021-00474-8

**Published:** 2021-03-16

**Authors:** Louis A. Chalmers, Samuel D. Searle, Jon Whitby, Alex Tsui, Daniel Davis

**Affiliations:** 1 Department of Population Science and Experimental Medicine, MRC Unit for Lifelong Health and Ageing at UCL, London, UK; 2 Department of Medicine, Dalhousie University, Halifax, NS Canada

**Keywords:** Delirium, Mortality, Survival, Aetiology, Ageing, Hospital

## Abstract

**Purpose:**

To describe aetiology-specific associations with mortality among older hospital patients with delirium.

**Methods:**

Over 21 months, a cohort of 1702 patients with 2471 acute hospital admissions (median age 85, IQR 80–90, 56% women) were assessed for delirium, categorised with inflammatory and metabolic aetiologies based on available laboratory results, and followed up for all-cause mortality. Interactions between aetiology and delirium were tested.

**Results:**

The total mortality for the cohort was 35.2%. While inflammation, metabolic disturbance, and delirium at time of admission all demonstrated independent associations with mortality, there was no evidence for any interactions between delirium and these laboratory-measured aetiologies.

**Conclusions:**

Delirium remains an important predictor of death in older hospital patients, irrespective of underlying aetiology.

## Key points

**Aim:**

To investigate aetiology-specific associations with mortality among older patients with delirium.

**Findings:**

Delirium predicted mortality, as did inflammatory and metabolic disorders. However, there was no evidence for any interactions between these factors.

**Message:**

Mortality from delirium is consistent regardless of underlying aetiology, suggesting that no aetiology carries better or worse prognosis than another.

## Introduction

Delirium, characterised by acute inattention and altered arousal, is common and serious in older inpatients [1]. It is associated with adverse outcomes including mortality, prolonged hospital stay, institutionalisation, and accelerated cognitive decline [2, 3]. Delirium aetiology is often multifactorial, though it is unclear whether the precipitating cause of delirium directly influences mortality. In order to address this question, we sought to quantify the relationship between different causes of delirium and mortality in a longitudinal cohort of older hospital patients.

## Methods

### Participants

We undertook a secondary analysis of a cohort presenting to an acute geriatric medicine service, previously described [4]. In brief, between April 2015 and January 2017, consecutive patients were assessed for frailty, dementia and delirium during the first 24 h of admission to a single tertiary centre, University College Hospital, London, which ran a parallel admissions service for older people based on frailty. Patients were included if they were admitted under this service for ≥ 24 h and had contemporaneous blood tests as part of their clinical work-up. Information from each admission was used if participants were hospitalised more than once. These analyses were conducted as part of a service evaluation project, and individual consent was not necessary as determined by the NHS Health Research Authority (HRA), the regulatory body for medical research for England, UK. The HRA has the Research Ethics Service as one of its core functions, and they determined the project was exempt from the need to obtain approval from an NHS Research Ethics Committee.

### Outcomes

Mortality was ascertained through statutory reporting (notifications to the National Health Service Spine registry). We considered outcomes up until December 2018.

### Exposures

Delirium was determined on initial presentation by a consultant geriatrician, often using a validated diagnostic tool such as the 4AT [5]. Dementia was generally diagnosed or identified by consultant geriatricians from medical records or collateral history at the time of admission. Frailty was graded according to the Clinical Frailty Scale [6].

Delirium aetiology was classified into four categories: inflammation, metabolic, neither, both. These categories were inferred from laboratory results and were also applied to non-delirious controls. Systemic inflammation was defined by an abnormal C-reactive protein (> 5 mg/L) or White Cell Count (< 4 or > 12 × 10^9^/L). Metabolic disturbance was defined by a high blood urea nitrogen/creatinine ratio (> 18 mg/dL:mg/dL) in the context of high creatinine (> 92 µmol/L) or by any other electrolyte abnormality (sodium < 135 or > 145 mmol/L; potassium < 3.5 or > 5.1 mmol/L, calcium (albumin-adjusted) < 2.15 or > 2.55 mmol/L). Such data were readily available from retrospective interrogation of electronic patient records.

### Statistical analysis

We used a proportional Cox hazard model to estimate risk of death in those with and without delirium, stratified by aetiology as defined above. To account for individuals with multiple admissions, we right-censored by readmission date [7] and clustered by individual to estimate robust standard errors. In a series of univariable and multivariable models, we tested interactions between delirium and each available aetiological factor, including the above exposures and demographic data (age, sex). Heteroskedasticity was assessed by inspecting Schoenfeld residuals. We used Python 3.6, Jupyter Notebook [8, 9], to pre-process the data and Stata 15.1 (StataCorp) for all other analyses.

## Results

In this sample, 1750 individuals were assessed. With 31% being admitted more than once, there were 2552 separate admissions. Inclusion criteria were met for 2471 admissions (96.8%, 1702 individual patients, 97.2%) (Table 1). Of these admissions, the median age was 85 years (range 52–103), 1378 (55.8%) were women, 1235 (50%) had dementia, and median Clinical Frailty Scale score was 6 (IQR 5, 7). Delirium was identified in 831 (33.6%) admissions. Inflammation and metabolic abnormalities were apparent in 1948 (78.8%) and 772 (31.2%) admissions, respectively. There were 866 (35.0%) deaths during the 44-month study period. Length of follow-up ranged from 1 to 1340 days.

**Table 1:** Patient characteristics and delirium status for admissions

	Overall (*n* = 2471)	No delirium (*n* = 1640)	Delirium (*n* = 831)	*p*
Mortality, *n* (%)	870 (35.2)	499 (30.4)	371 (44.6)	< 0.001
Age, median (Q1,Q3)	85 [81,90]	85 (81,89)	85 (80,90)	0.745
Sex, female (%)	1378 (55.8)	914 (55.7)	464 (55.8)	0.995
1	2 (0.1)	2 (0.1)	0 (0)	
2	9 (0.4)	9 (0.5)	0 (0)	
3	32 (1.3)	31 (1.9)	1 (0.1)	
4	170 (6.9)	138 (8.4)	32 (3.9)	
Clinical Frailty Score (%)				
5	602 (24.4)	473 (28.8)	129 (15.5)	< 0.001
6	912 (36.9)	603 (36.8)	309 (37.2)	
7	622 (25.2)	325 (19.8)	297 (35.7)	
8	95 (3.8)	44 (2.7)	51 (6.1)	
9	27 (1.1)	15 (0.9)	12 (1.4)	
Dementia (%)	1235 (50.0)	626 (38.2)	609 (73.3)	< 0.001
Inflammatory (%)	1947 (78.8)	1248 (76.1)	700 (84.2)	< 0.001
Metabolic (%)	772 (31.2)	490 (29.9)	282 (33.9)	0.044

**Fig. 1:**
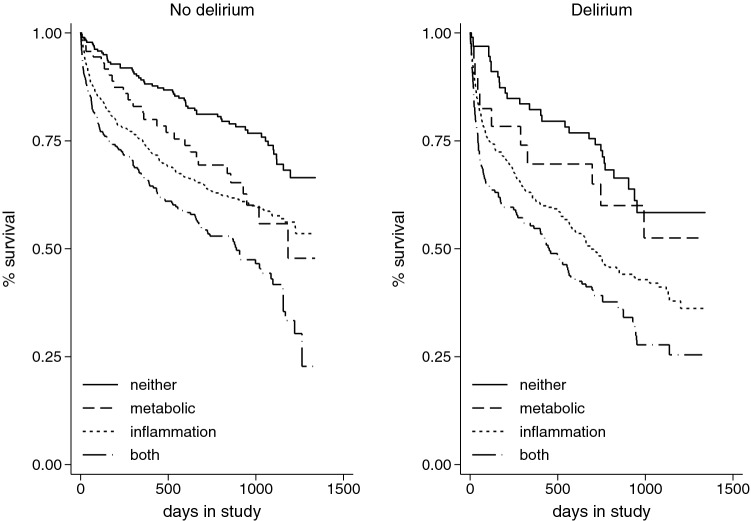
Kaplan–Meier survival curves describing age–sex–frailty-adjusted survival from admission, by aetiology and delirium status (censored by readmission or end of study, clustered by patient)

In univariable models, delirium, inflammation and metabolic disturbance were all associated with mortality (delirium HR 1.35, 95% CI 1.17–1.56, *p* < 0.01; inflammation HR 1.74, 95% CI 1.45–2.09, *p* < 0.01; metabolic HR 1.36, 95% CI 1.18–1.56, *p* < 0.01) (Table 2). Kaplan–Meier curves, adjusted by age–sex–frailty, show survival stratified by delirium status and aetiology (Fig. 1). In the multivariable model, there was no evidence of an interaction between delirium status and aetiological factor (Table 2).

**Table 2:** Hazard ratios for mortality by delirium, inflammatory and metabolic exposures, and covariate factors, estimated by Cox regression

	Univariable models				Multivariable model			
	HR	95% CI		*p*	HR	95% CI		*p*
Age	1.04	1.03	1.05	< 0.01	1.03	1.02	1.04	< 0.01
Sex (female)	0.73	0.63	0.83	< 0.01	0.72	0.64	0.83	< 0.01
Clinical Frailty Score	1.45	1.34	1.56	< 0.01	1.43	1.33	1.54	< 0.01
Dementia	0.92	0.79	1.07	0.28				
Delirium	1.35	1.17	1.56	< 0.01	1.32	1.15	1.52	< 0.01
Inflammatory	1.74	1.45	2.09	< 0.01	1.76	1.47	2.11	< 0.01
Metabolic	1.36	1.18	1.56	< 0.01	1.36	1.18	1.57	< 0.01

Complete case analysis of 2471 admissions, clustered by 1702 patients

## Discussion

In a sample of older people presenting to acute care, we found the mortality associated with delirium was consistent, irrespective of underlying inflammatory, metabolic or other aetiologies. This suggests any pathophysiological pathway from delirium to death does not specifically involve inflammation or metabolic disturbance. It also indicates the mortality after delirium is substantial, even if there are no abnormal laboratory results. Taken together, our findings highlight the need for continued recognition of delirium as a harbinger for death from any cause.

Consistent with the case mix for an acute frailty service (> 90% had CFS scores 5 or more), admissions with delirium were of comparable age to those without. Similarly, dementia was not associated with death on univariable analysis, compared with frail patients without dementia. These observations are in keeping with our finding that adjusting for age did not make much difference between univariable and multivariable analyses.

Our results are limited as a single-site study in an urban university hospital. Moreover, we have assumed that routine laboratory tests are valid approaches to classify systemic inflammatory or metabolic aetiologies. Additional information on medications as delirium precipitants may have strengthened our interpretation. Nonetheless, the data benefit from prospective follow-up of a cohort characterised by specialists in geriatric medicine, and the outcome ascertainment is robust.

Other studies that have linked aetiology-based phenotypes in delirium with outcomes such as cognitive impairment, have been mainly in the context of critical illness or after surgery [10–12]. In these settings, specific delirium aetiologies may have different impacts on outcomes. This contrasts with our finding that delirium carries similar mortality outcomes, regardless of aetiology.

Our findings emphasise the impact of delirium on mortality irrespective of aetiology for older patients presenting to acute hospital. The main clinical implication is that delirium, even when its precipitant does not affect laboratory values (e.g. new medications or withdrawal, pain, constipation, urinary retention), confers an association with mortality comparable to other causes that may be perceived as more serious. Further work is needed to confirm this in other settings, such as intensive care or postoperative patients. Mechanistically, if the causes considered here do not have specific relationships with death, that is, any aetiology is equally relevant, then it suggests that delirium represents a final common (adverse) pathway. In any case, persistent effort is needed to ensure best practice in delirium prevention but also that identifying all causes of delirium remains an important aspect of geriatric medical care.

## Data Availability

On request subject to data sharing agreement (https://www.ucl.ac.uk/cardiovascular/research/population-science-and-experimental-medicine/mrc-unit-lifelong-health-and-ageing-ucl/data).

## Code availability

On request.

## References

[1] Gibb K, Seeley A, Quinn T, Siddiqi N, Shenkin S, Rockwood K, Davis D (2020). The consistent burden in published estimates of delirium occurrence in medical inpatients over four decades: a systematic review and meta-analysis study. Age Ageing.

[2] Witlox J, Eurelings LS, de Jonghe JF, Kalisvaart KJ, Eikelenboom P, van Gool WA (2010). Delirium in elderly patients and the risk of postdischarge mortality, institutionalization, and dementia: a meta-analysis. JAMA.

[3] Davis DH, Muniz-Terrera G, Keage HA, Stephan BC, Fleming J, Ince PG, Matthews FE, Cunningham C, Ely EW, MacLullich AM, Brayne C, Epidemiological Clinicopathological Studies in Europe (EClipSE) Collaborative Members (2017). Association of delirium with cognitive decline in late life: a neuropathologic study of 3 population-based cohort studies. JAMA Psychiatry..

[4] Ellis HL, Wan B, Yeung M, Rather A, Mannan I, Bond C, Harvey C, Raja N, Dutey-Magni P, Rockwood K, Davis D, Searle SD (2020). Complementing chronic frailty assessment at hospital admission with an electronic frailty index (FI-Laboratory) comprising routine blood test results. CMAJ.

[5] References [Internet]. 4AT—rapid clinical test for delirium [cited 2020 Aug 10]. https://www.the4at.com/references

[6] Rockwood K, Song X, MacKnight C, Bergman H, Hogan DB, McDowell I, Mitnitski A (2005). A global clinical measure of fitness and frailty in elderly people. CMAJ.

[7] Prinja S, Gupta N, Verma R (2010). Censoring in clinical trials: review of survival analysis techniques. Indian J Community Med.

[8] Kluyver T, Ragan-Kelley B, Pérez F, Granger B, Bussonnier M, Frederic J et al (2016) Jupyter Notebooks—a publishing format for reproducible computational workflows. In: Loizides F, Scmidt B (eds) Positioning and power in academic publishing: players, agents and agendas [Internet]. IOS Press, pp 87–90. https://eprints.soton.ac.uk/403913/

[9] Pollard TJ, Johnson AEW, Raffa JD, Mark RG (2018). tableone: an open source Python package for producing summary statistics for research papers. JAMIA Open.

[10] Girard TD, Thompson JL, Pandharipande PP, Brummel NE, Jackson JC, Patel MB, Hughes CG, Chandrasekhar R, Pun BT, Boehm LM, Elstad MR, Goodman RB, Bernard GR, Dittus RS, Ely EW (2018). Clinical phenotypes of delirium during critical illness and severity of subsequent long-term cognitive impairment: a prospective cohort study. Lancet Respir Med.

[11] Page VJ (2018). Does sedation related delirium matter?. Lancet Respir Med.

[12] Canet E, Amjad S, Robbins R, Lewis J, Matalanis M, Jones D, Bellomo R (2019). Differential clinical characteristics, management and outcome of delirium among ward compared with intensive care unit patients. Intern Med J.

